# Disruption of the Golgi Apparatus and Contribution of the Endoplasmic Reticulum to the SARS-CoV-2 Replication Complex

**DOI:** 10.3390/v13091798

**Published:** 2021-09-09

**Authors:** Ted Hackstadt, Abhilash I. Chiramel, Forrest H. Hoyt, Brandi N. Williamson, Cheryl A. Dooley, Paul A. Beare, Emmie de Wit, Sonja M. Best, Elizabeth R. Fischer

**Affiliations:** 1 Host-Parasite Interactions Section, Laboratory of Bacteriology, Rocky Mountain Laboratories, National Institute of Allergy and Infectious Diseases, National Institutes of Health, Hamilton, MT 59840, USA; cdooley@niaid.nih.gov; 2 Innate Immunity and Pathogenesis Section, Laboratory of Virology, Rocky Mountain Laboratories, National Institute of Allergy and Infectious Diseases, National Institutes of Health, Hamilton, MT 59840, USA; abhilash.chiramel@gmail.com (A.I.C.); sbest@niaid.nih.gov (S.M.B.); 3 Microscopy Unit, Research Technologies Branch, Rocky Mountain Laboratories, National Institute of Allergy and Infectious Diseases, National Institutes of Health, Hamilton, MT 59840, USA; forrest.hoyt@nih.gov (F.H.H.); EFISCHER@niaid.nih.gov (E.R.F.); 4 Molecular Pathogenesis Unit, Laboratory of Virology, Rocky Mountain Laboratories, National Institute of Allergy and Infectious Diseases, National Institutes of Health, Hamilton, MT 59840, USA; Brandi.Williamson@nih.gov (B.N.W.); emmie.dewit@nih.gov (E.d.W.); 5 Coxiella Pathogenesis Section, Laboratory of Bacteriology, Rocky Mountain Laboratories, National Institute of Allergy and Infectious Diseases, National Institutes of Health, Hamilton, MT 59840, USA; pbeare@niaid.nih.gov

**Keywords:** SARS-CoV-2, cell biology, microscopy, electron microscopy, Golgi, ER

## Abstract

A variety of immunolabeling procedures for both light and electron microscopy were used to examine the cellular origins of the host membranes supporting the SARS-CoV-2 replication complex. The endoplasmic reticulum has long been implicated as a source of membrane for the coronavirus replication organelle. Using dsRNA as a marker for sites of viral RNA synthesis, we provide additional evidence supporting ER as a prominent source of membrane. In addition, we observed a rapid fragmentation of the Golgi apparatus which is visible by 6 h and complete by 12 h post-infection. Golgi derived lipid appears to be incorporated into the replication organelle although protein markers are dispersed throughout the infected cell. The mechanism of Golgi disruption is undefined, but chemical disruption of the Golgi apparatus by brefeldin A is inhibitory to viral replication. A search for an individual SARS-CoV-2 protein responsible for this activity identified at least five viral proteins, M, S, E, Orf6, and nsp3, that induced Golgi fragmentation when expressed in eukaryotic cells. Each of these proteins, as well as nsp4, also caused visible changes to ER structure as shown by correlative light and electron microscopy (CLEM). Collectively, these results imply that specific disruption of the Golgi apparatus is a critical component of coronavirus replication.

## 1. Introduction

Coronaviruses are widespread in nature with thousands of genomes being annotated. Only a very few are known to be pathogenic toward humans and had been associated primarily with mild upper respiratory infections such as common colds [1]. In 2002, SARS-coronavirus apparently crossed species barriers to cause Severe Acute Respiratory Syndrome (SARS) in humans. Although the disease had a high mortality, symptoms appeared prior to patients becoming highly infectious thus dissemination was inefficient enough that public health measures silenced the outbreak. A similar scenario occurred in 2012 with Middle East Respiratory Syndrome (MERS) coronavirus, which also showed a high mortality rate but was again contained by public health measures. SARS is no longer epidemic in humans and it is unclear whether the virus remains in animal reservoirs. MERS, however, still occasionally spills over from camels to cause limited outbreaks. In late 2019, another coronavirus emerged in Wuhan, China [2]. In this case, mortality rates were lower, however, asymptomatic cases were common and the virus was transmissible prior to the onset of symptoms. The virus was communicated very efficiently and the disease quickly progressed to a global pandemic. This virus was named SARS-CoV-2 and the associated disease COVID19. The overall mortality rate of COVID19 is approximately equivalent to the 1918 influenza pandemic [3]. Although many coronavirus genomes have been annotated, the pathogenic potential of most of these is unknown. There is thus a high likelihood of future outbreaks.

Coronaviruses are enveloped, positive (+)-strand RNA viruses. Following fusion of the viral and host membranes to release viral genomic RNA, 29 viral proteins are expressed [4]. Like all positive-strand RNA viruses including picornaviruses, noroviruses, hepatitis C virus, and arteriviruses, replication of the coronavirus genome occurs in association with membranous replication organelles [5,6,7,8,9]. Extensive rearrangements of cellular membranes contribute to the creation of the replication complexes. Advanced electron microscopy technologies have contributed greatly to our understanding of these membranous structures. Although ER is most frequently subverted in the creation of these viral replication organelles [10], multiple cellular sources of membrane, such as the Golgi apparatus, mitochondrial outer membrane, or plasma membrane, may contribute to the biogenesis of these complexes. For recent reviews, please see Wolff et al., [7] and Harak and Lohmann [11]. The replication organelles are believed to concentrate host and viral factors necessary for viral RNA synthesis and may also sequester replication intermediates from the innate immune system [12].

## 2. Material and Methods

### 2.1. Virus and Cell Lines

SARS-CoV-2 isolate nCoV-WA1-2020 (MN985325.1) [13] (Vero passage 3) was kindly provided by CDC and propagated once in VeroE6 cells in DMEM (Sigma, Fairfax, VA, USA) supplemented with 2% fetal bovine serum (Gibco, Grand Island, NY, USA), 1 mM L-glutamine (Gibco), 50 U/mL penicillin and 50 μg/mL streptomycin (Gibco). Virus titrations were performed by end-point titration in Vero E6 cells as previously described [14]. Cells were inoculated with 10-fold serial dilutions of samples. One hour after inoculation of cells, the inoculum was removed and replaced with 100 µL virus isolation medium. Six days after inoculation, CPE was scored and the TCID50 was calculated. For some experiments, ACE2-expressing A549 human lung epithelial cells were used and propagated in DMEM with 5% fetal bovine serum.

### 2.2. Antibodies

Anti-SARS-CoV-2 Spike antibody, anti-nucleocapsid antibody, and anti-nucleoprotein antibody were from ProSci, Inc. (Poway, CA, USA). Anti-SARS-CoV-2 anti-Membrane (M) glycoprotein antibody was a custom synthesized rabbit polyclonal antibody to the peptide: KLNDTHSSSSDNIALLVQ (Thermo Fisher Scientific/Invitrogen, Waltham, MA, USA). Anti-TGN46, giantin, GM130, and calnexin were from Abcam (Cambridge, UK). Anti-TGN46 from Thermo Fisher Scientific/Invitrogen, Waltham, MA, USA was used in some experiments. Anti-Strep tag was from Sigma. Monoclonal antibody SCICONS J2 to dsRNA was from English and Scientific Consulting, Kft, Hungary. Secondary antibodies, anti-mouse Ig or anti-rabbit Ig, were Fab’-fragments conjugated to DyLight_488_ or DyLight_594_ or horseradish peroxidase (Jackson ImmunoResearch, West Grove, PA, USA). All primary antibodies and fluorescent secondary antibodies were used at a dilution of 1:200. Anti-horseradish peroxidase secondary antibody was used at 1:100.

*Helix pomatia* agglutinin (HPA) lectin conjugated to AlexaFluor 488 was from Invitrogen and used at a final concentration of 5 μg/mL. Vital staining with C6-NBD-ceramide (Thermo Fisher Scientific/ Invitrogen, Waltham, MA, USA) was performed as previously described [15].

### 2.3. Immunofluorescence

Vero cells were infected with a calculated MOI of up to 5 based upon TCID50. Maximum number of infected cells, however, never exceeded approximately 70%. Infected cultures on 12 mm glass coverslips were fixed at the stated times in 2% paraformaldehyde for 24 h at room temperature, rinsed once with 50 mM NaPO_4_, 150 mM NaCl, pH 7.4 buffer (PBS), permeabilized with PBS plus 0.1% TX-100, and labeled with primary antibodies for 1hat room temperature. The coverslips were rinsed with PBS and incubated with the respective secondary antibody for 1 hr. Images were acquired on a Nikon Eclipse 80i microscope with a 60× 1.4-numerical aperture oil immersion objective and a Nikon DS-Qi1Mc camera using Nikon Elements AR 3.2 for image capture. Images were processed for figures using Adobe Photoshop 22.4.3. Representative images are shown.

### 2.4. Ectopic Expression of SARS-CoV-2 Proteins

A library of annotated SARS-CoV-2 ORFs and mature NSPs in the expression vector pLVX-EF1alpha-IRES-Puro (Takara Bio USA, San Jose, CA, USA) [4] was kindly provided by N.J. Krogan. A gBlock^TM^ gene fragment containing an N-terminal 2xStrep tag fused to the nsp3 gene sequence was constructed by Integrated DNA Technologies. This fragment was inserted by In-Fusion HD cloning (Takara) into EcoRI/BamHI digested pLVX-EF1a-IRES-Puro plasmid.

Constructs were transfected into Vero cells using a Lipofectamine LTX Plus Kit (Invitrogen, Life Technologies, Carlsbad, CA, USA) according to the manufacturer’s instructions. Cells were fixed 24 h post transfection with paraformaldehyde and processed for immunofluorescence analysis as described above.

### 2.5. Transmission Electron Microscopy

Specimens grown on Thermanox™ (Ted Pella, Redding, CA, USA) coverslips were fixed with 2% paraformaldehyde/2.5% glutaraldehyde in 0.1 M Sorenson’s phosphate buffer, and then post-fixed with 1.0% osmium tetroxide/0.8% potassium ferricyanide in 0.1 M sodium cacodylate buffer, washed then stained with 1% tannic acid in dH_2_O. The samples were further osmicated with 2% osmium tetroxide in 0.1M sodium cacodylate, then washed with dH_2_O and additionally stained overnight with 1% uranyl acetate at 4 °C (Ted Pella, Redding, CA, USA). After washing with dH_2_O, specimens were dehydrated with a graded ethanol series, and embedded in Spurr’s resin. Thin sections were cut with a Leica UC7 ultramicrotome (Buffalo Grove, IL, USA) prior to viewing at 120 kV on a FEI BT Tecnai transmission electron microscope (Thermo Fisher/FEI, Hillsboro, OR, USA). Digital images were acquired with a Gatan Rio camera (Gatan, Pleasanton, CA, USA).

### 2.6. Focused Ion Beam/Scanning Electron Microscopy

Samples were fixed in 2.5% glutaraldehyde in 0.1 M sodium cacodylate. Post fixation was performed with 0.5% osmium tetroxide/0.8% sodium ferricyanide in 0.1 M sodium cacodylate buffer. The samples were then washed in 0.1 M sodium cacodylate buffer, stained with 1% tannic acid, washed, and then osmicated again with 1% osmium tetroxide in 0.1 M sodium cacodylate. The samples were then washed in water then stained with 1% uranyl acetate. Samples were dehydrated in a graded ethanol exchange and embedded in EPON/Araldite. The blocks were trimmed and attached to aluminum SEM stubs. To improve the sample conductivity a thin layer of carbon was applied to the stubs using a 208-Carbon Cressington Carbon Coater (Cressington, Watford, UK). Focused ion beam scanning electron microscopy was performed using a Helios G4 UX (Thermo Fisher Scientific/FEI Hillsboro, OR, USA). Areas of interest were milled with a focused gallium ion beam at 30 kV and 2.4 µA to remove 10 nm thick slices. The sequentially exposed block face was imaged at 2 kV and 0.8 nA with a dwell time of 3 µs using the In-Column Detector. Image stacks were aligned using IMOD [16]. Features of interest were segmented using the convolutional neural network-based annotation implemented in EMAN2 [17]. Hand segmentation was performed using Amira (Thermo Fisher Scientific/FEI Hillsboro, OR, USA).

### 2.7. Immunoelectron Microscopy

Vero cells were grown on Thermanox™ (Ted Pella, Redding, CA, USA) coverslips and infected SARS-CoV-2 and incubated for the stated times. Cells were rinsed with Hanks balanced salt solution, followed by fixation with 2% paraformaldehyde overnight at room temperature. Specimens were rinsed with PBS, permeabilized with 0.01% saponin in PBS for 5 min at room temperature and incubated with primary antibodies followed by peroxidase conjugated secondary antibody in 0.01% saponin in PBS. Samples were fixed for 1 h with 1.5% glutaraldehyde in 0.1 M sodium-cacodylate with 5% sucrose (pH 7.4), rinsed three times and developed using the Pierce diaminobenzidine (DAB) metal-enhanced substrate kit prior to embedding. Samples were embedded in Spurr’s resin (Ted Pella, Redding CA, USA) and micrographs were acquired using a 120 kV FEI BT Tecnai transmission electron microscope (Thermo Fisher/FEI, Hillsboro, OR, USA). Digital images were acquired with a Gatan Rio camera (Gatan, Pleasanton, CA, USA).

### 2.8. Correlative Light and Electron Microscopy (CLEM)

Cells were grown and transfected on Bellco photo etched coverslips (Bellco Glass, Vineland, NJ, USA). Transfected cells were identified by immunofluorescence using an anti-strep-tag antibody and images collected for regions of interest using a Evos FL Auto 2 microscope (Thermo Fisher/FEI, Hillsboro, OR, USA). The cells were then processed for TEM imaging as described above. Using the gridding imprinted into the block face by the coverslips, areas of interest were reidentified and aligned using the MAPS Software (Thermo Fisher/FEI, Hillsboro, OR, USA). The blocks were trimmed close to the ROI and sections collected at 70 nm using a Leica UC6 ultramicrotome (Leica Microsystems, Buffalo Grove, IL, USA). TEM micrographs were collected using a Hitachi HT7800 (Hitachi, Tokyo, Japan) and the final images were correlated with the fluorescent images using MAPS (Thermo Fisher/FEI, Hillsboro, OR, USA). Depending upon transfection efficiency, numbers of transfected cells examined ranged from a minimum of three to over 20. Representative images are shown.

## 3. Results

### 3.1. Kinetics of SARS-CoV-2 Replication Complex Formation

**Figure 1:**
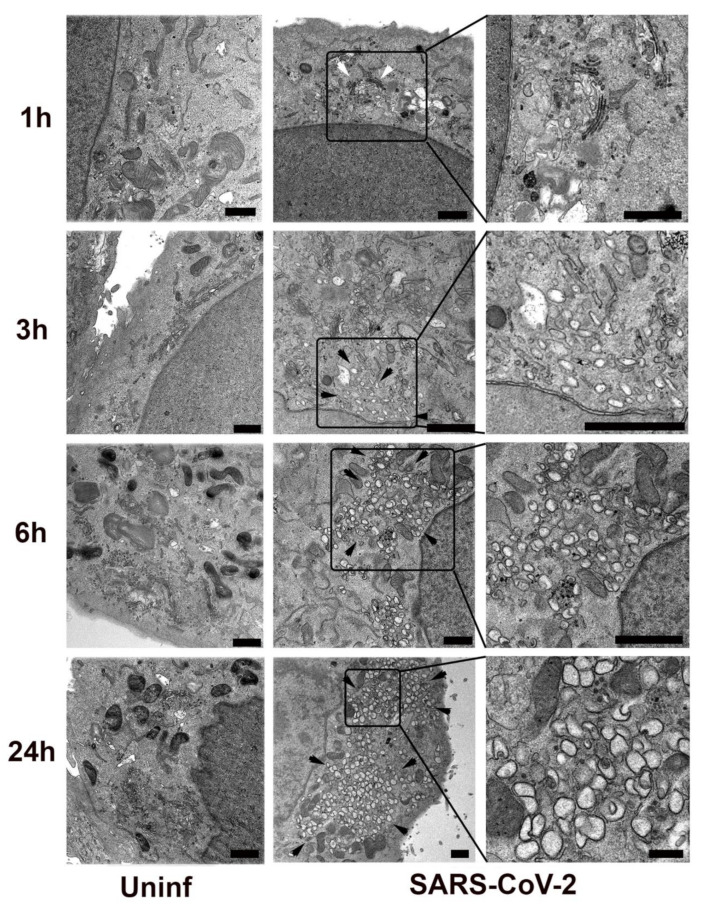
SARS-CoV-2 infected or mock-infected (Uninf) Vero E6 cells were fixed and processed for transmission electron microscopy at 1, 3, 6, or 24 h post-infection. Black arrowheads indicate regions enriched in SARS-CoV-2 induced membrane proliferation. White arrowhead indicates the intact Golgi apparatus in the 1 h sample. Delineated regions of interest in the infected cells are enlarged in the right column to show detail. Bars = 1 μm.

We first examined the kinetics of SARS-CoV-2 replication organelle formation in Vero E6 cells (Figure 1). By 3 h post-infection, vacuolization was observed in a perinuclear region with multiple vesicles approx. 50–150 nm in diameter. By 6 h post-infection, these vesicles had increased in size and number, showing the typical 250–500 nm diameter double membrane vesicles (DMVs) characteristic of coronavirus replication organelles. These vesicles further increased in number and complexity and by 24 h post-infection occupied a large part of the cytosol.

**Figure 2:**
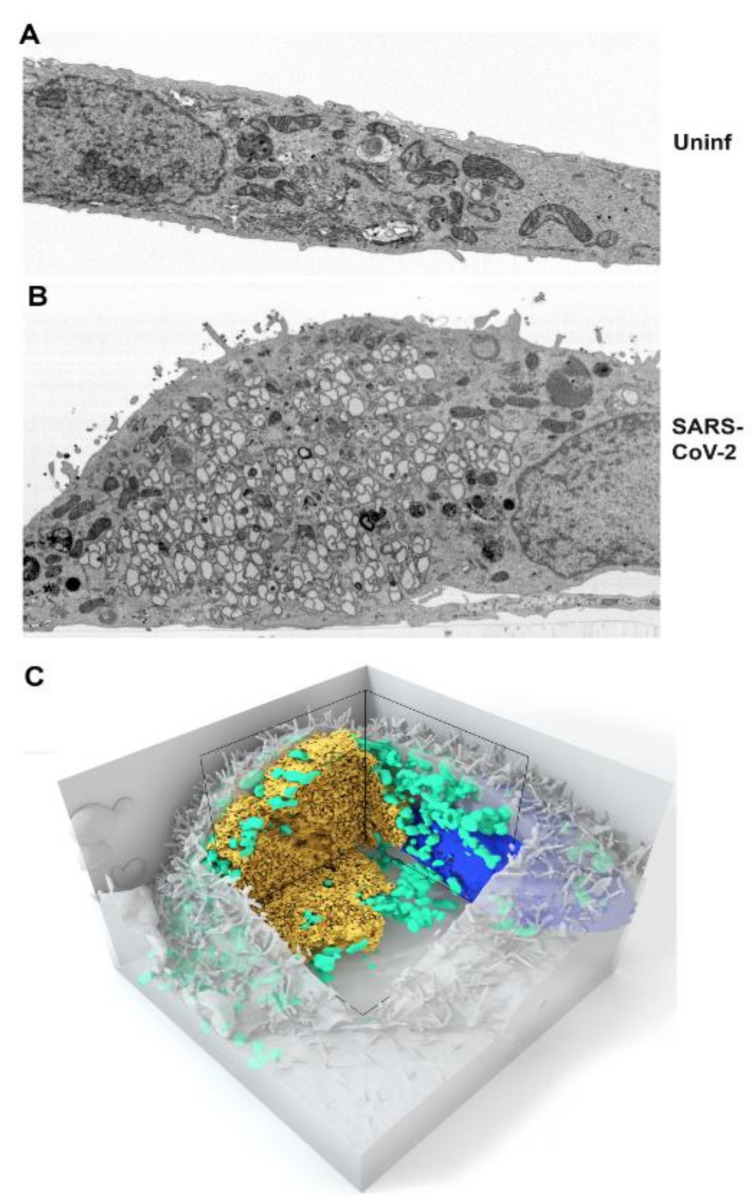
Vero E6 cells were mock-infected (**A**) or SARS-CoV-2 infected (**B**) and fixed at 24 h post-infection for Focused Ion Beam-Scanning Electron Microscopy (FIB-SEM). Single slices are shown in panels (**A**,**B**). The full image stacks are shown in Supplemental Video S1. (**C**) 3D volume rendering of the SARS-CoV-2 infected cell depicted in panel B and the Supplemental Movie Video S1. The replication complex is depicted in gold. Mitochondria are shown in green and the nucleus in blue.

Focused Ion Beam-Scanning Electron Microscopy (FIB-SEM) was used to give a 3-dimensional view of the replication organelle. A single slice of a mock- and SARS-CoV-2-infected Vero cells is depicted in Figure 2A,B. The full image stack video is provided as Supplemental Video S1. Similar to a recent publication using the same advanced imaging technology [18], complex interconnected membrane structures were visible and contiguous with presumed ER derived membrane based upon ultrastructure. Mitochondria appeared displaced by the replication complex. A 3D rendering demonstrating the accumulation of mitochondria at the periphery of the replication complex is shown in Figure 2C. While it is likely that the size of the replication complex displaces mitochondria, the adjacent cytosolic regions appear devoid of mitochondria suggesting the possibility of a more active mechanism for mitochondrial recruitment.

### 3.2. SARS-CoV-2 Structural Protein Synthesis and Localization

SARS-CoV-2 structural protein appearance and localization was similarly examined over a 24 h time course (Figure S1). Viral nucleocapsid protein (N) was regularly detected in a punctate perinuclear location as early as 3h post-infection. By 6 h post-infection, the N protein appears evenly distributed throughout the infected cell, however, by 12 h and later post-infection, it was frequently observed enriched around large, membranous organelles possibly representing the replication complex. In contrast, neither the spike protein (S) nor the membrane glycoprotein (M) was detected until 6 h post-infection when they were observed in a peri-nuclear location. The S protein remained enriched in the peri-nuclear region throughout the observation period and often associated with the nuclear envelope. In contrast, the M protein typically appeared more dispersed by 12 h and later post-infection.

**Figure 3:**
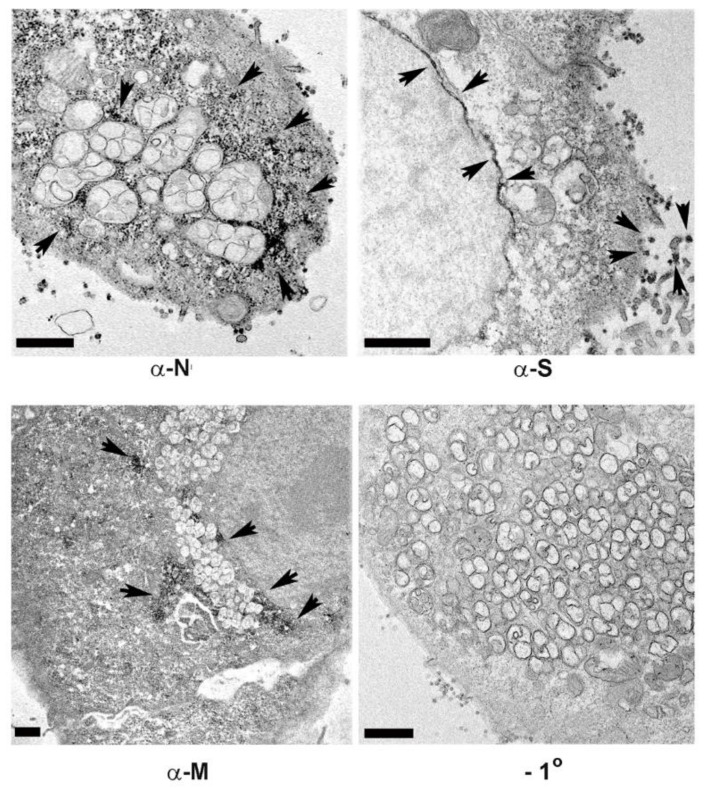
Immunoelectron microscopy localization of viral structural antigens in Vero E6 cells fixed at 24 h post-infection for horseradish peroxidase secondary antibody labeling with diaminobenzidine staining. Dark reaction product indicates localization of the antigen. Shown are anti-nucleocapsid (N), Spike protein (S), and Membrane glycoprotein (M). A panel showing negative staining in the absence of the primary antibody is shown (−1°). Arrowheads indicate regions of HRP-DAB labeling. Bars = 1 μm.

Immunoelectron microscopy (IEM) of cells 24 h post-infection using horseradish peroxidase with metal enhanced diaminobenzidine (HRP-DAB) detection demonstrated enrichment of the N protein around membrane encapsulated vesicles of the replication complex. Consistent with the immunofluorescence staining, the S protein was enriched in the nuclear envelope and also observed on extracellular virions (Figure 3). The M protein was observed in discreet areas that were distributed throughout the replication complex.

**Figure 4:**
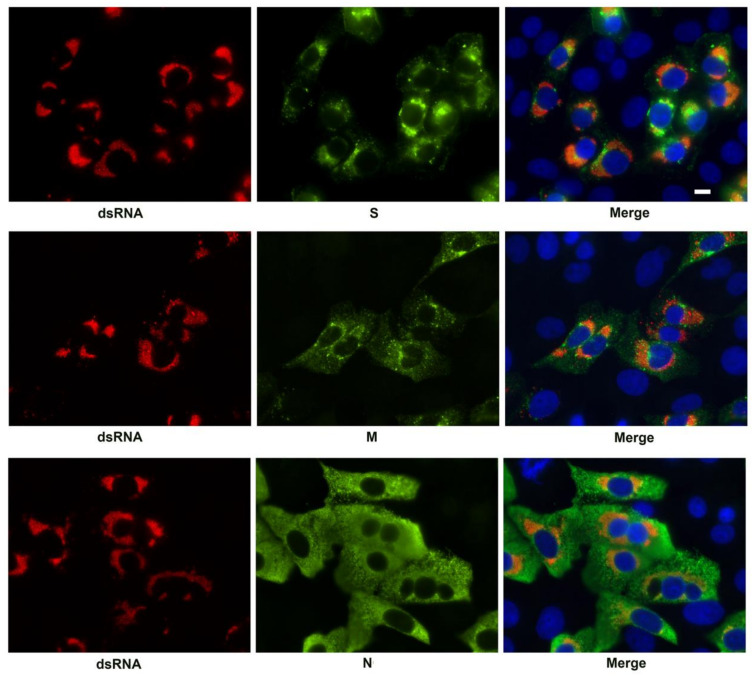
Immunofluorescent labeling of the SARS-CoV-2 replication complex at 24 h post-infection by antibody detecting dsRNA (red). Viral antigens S, M, and N, are shown in green. The merged image is counterstained with DAPI (blue). Bar = 10 μm.

Viral RNA occurs within DMVs of the SARS-CoV-2 replication complex [8]. We used antibodies against double stranded RNA (dsRNA) to identify the replication complexes and counterstained for the SARS-CoV-2 S, M, and N structural proteins at 24 h post infection. Although there were compact regions of intermixing of the S and M proteins with the dsRNA positive vesicles, no distinct co-staining was observed. In contrast, the N protein was present throughout the infected cells and therefore overlapped with the dsRNA but did not appear specifically enriched in association with these structures (Figure 4).

### 3.3. Effect of SARS-CoV-2 on Golgi Apparatus and ER Organization

**Figure 5:**
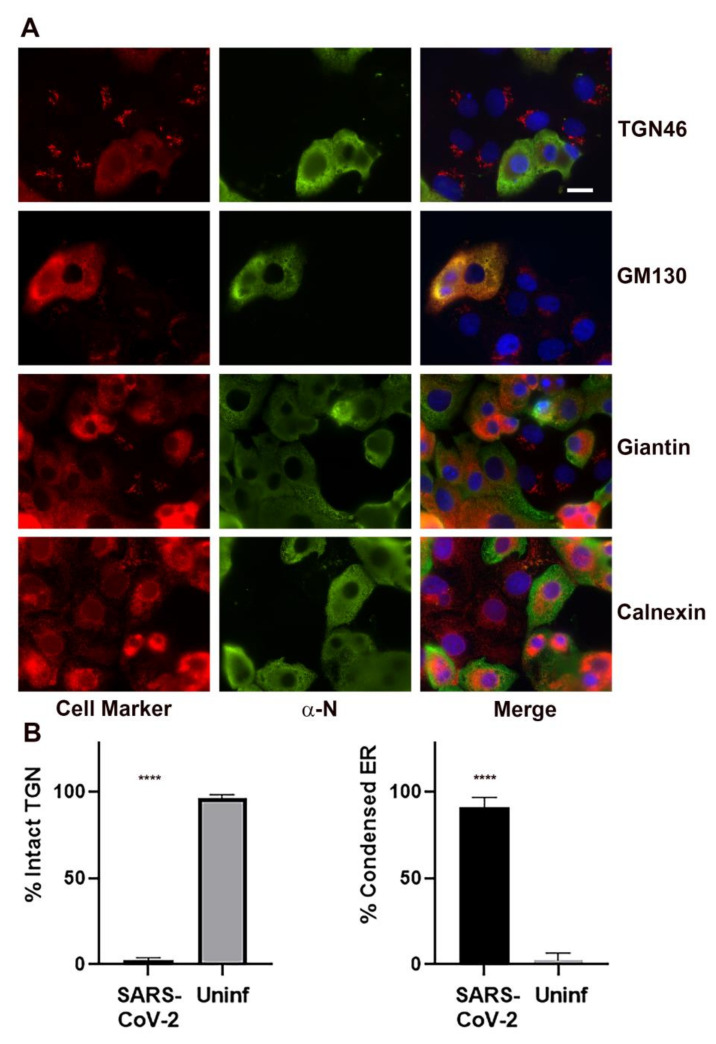
Immunofluorescent labeling for Golgi and ER markers in SARS-CoV-2 infected cells showing dissolution of the Golgi apparatus and compaction of the ER in infected cells. (**A**) Golgi specific markers include TGN46, GM130, and giantin. The ER was labeled with calnexin. Infected cells are labeled with an anti-nucleocapsid (N) antibody (green). Merged images are counterstained with DAPI (blue). Bar = 10 μm. (**B**) Quantitation of the TGN46 observed in cells infected with SARS-CoV-2 vs. uninfected control cells. TGN46 localization was determined for 315 SARS-CoV-2 infected cells in three biological replicates and 522 uninfected control cells in five biological replicates. For ER condensation, 125 SARS-CoV-2 infected cells and 106 uninfected control cells in five biological replicates were analyzed. Shown is the mean ± the S.E.M. Statistics were performed using One-way ANOVA. Significant differences relative to the uninfected control are indicated (**** *p* < 0.0001).

We examined several markers for cellular organelles at various stages of infection (Figure 5A). Notably, the Golgi apparatus was dramatically fragmented and dispersed in infected cells. Specific Golgi markers examined included TGN46, GM130, and giantin. Calnexin was used as a marker for the ER. The ER, widely thought to be a source of membrane for the coronavirus replication complex [7,8,18,19,20,21,22], appeared retracted and condensed in infected cells. Quantitation of the frequency of Golgi dispersal revealed that by 24 h post-infection, the Golgi apparatus was intact in 2.2 ± 0.8% of the infected cells compared to 96.6 ± 0.8% in the uninfected cells. The frequency of ER condensation was also quantified at 24 h post-infection. The ER was condensed in 90.2 ± 3.4% of the infected cells compared to 2.3 ± 2.3% condensed in the uninfected cells (Figure 5B).

We examined the kinetics of Golgi dispersal during SARS-CoV-2 infection and observed partial fragmentation of the Golgi apparatus by 6 h post-infection (Figure S2). By 12 h post-infection, the dispersal appeared complete. We also examined the effect of SARS-CoV-2 infection on Golgi disruption in ACE2-expressing A549 cells (Figure S3). The Golgi apparatus was similarly dispersed in this human lung derived epithelial cell line.

**Figure 6:**
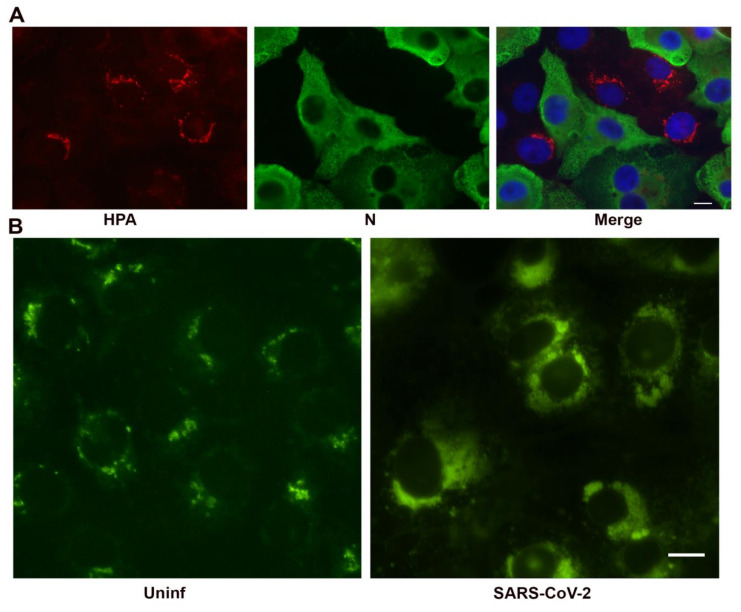
Non-antibody labeling of the Golgi apparatus in infected cells at 24 h post-infection. (**A**) *Helix pomatia* agglutinin labeling of the Golgi apparatus (red). Infected cells are shown in green and the nucleus in blue in the merged image. (**B**) C_6_-NBD-ceramide labeling of Golgi membrane in glutaraldehyde fixed, infected cells. Bars = 10 μm.

We verified fragmentation of the Golgi apparatus using non-antibody based methodology. SARS-CoV-2 infected Vero cells were labeled with the lectin *Helix pomatia* agglutinin (HPA), which recognizes immature glycoproteins transiting the Golgi apparatus [23,24]. The Golgi apparatus was dispersed in infected cells as seen with the antibody staining (Figure 6A). In an effort to demonstrate Golgi-derived lipids in association with the replication organelle, we labeled paraformaldehyde-fixed, SARS-CoV-2 infected Vero cells with C_6_-NBD-ceramide (Figure 6B), a ceramide analog used as a vital stain for the Golgi apparatus [25]. C_6_-NBD-ceramide labeling is not compatible with antibody labeling as permeabilization disrupts membranes and ceramide localization. However, the C_6_-NBD-ceramide was observed widely distributed in SARS-CoV-2 infected cultures in vacuolar structures resembling the replication complex suggesting that Golgi derived lipids contribute to formation of the replication organelle.

### 3.4. Association of Cellular Markers with the SARS-CoV-2 Replication Organelle

**Figure 7:**
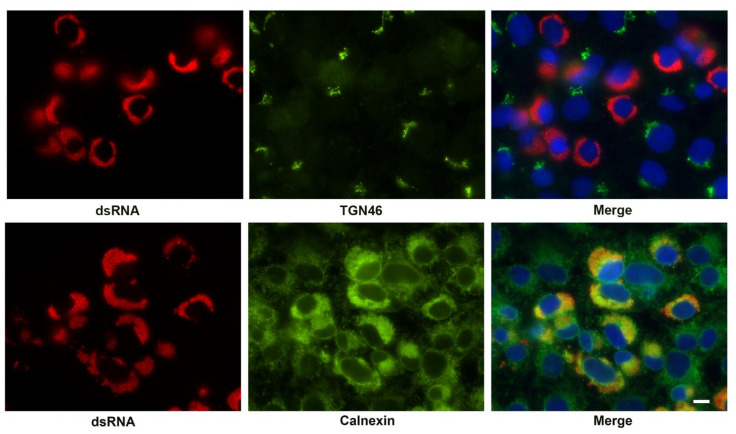
Immunofluorescent labeling of the SARS-CoV-2 replication complex at 24 h post-infection by antibody detecting dsRNA (red) and co-labeling with antibody to Golgi (TGN46) and ER markers (Calnexin). Cellular antigens are shown in green. The merged image is counterstained with DAPI (blue). Note the condensation of the ER and close association with the viral replication organelle. TGN46 appears only as diffuse background in infected cells. Bar = 10 μm.

We also examined the association of cellular markers for the Golgi apparatus (TGN46) and ER (calnexin) with the replication complex (Figure 7). While the Golgi apparatus was dispersed in SARS-CoV-2 infected cells, no specific enrichment around the replication organelles was observed. As shown above, the ER was condensed in the infected cells. Indeed, the ER appeared retracted from some areas of the cytosol as described. The condensed ER was the only viral or cellular marker observed that specifically associated with the replication organelle indicated by the presence of dsRNA.

**Figure 8:**
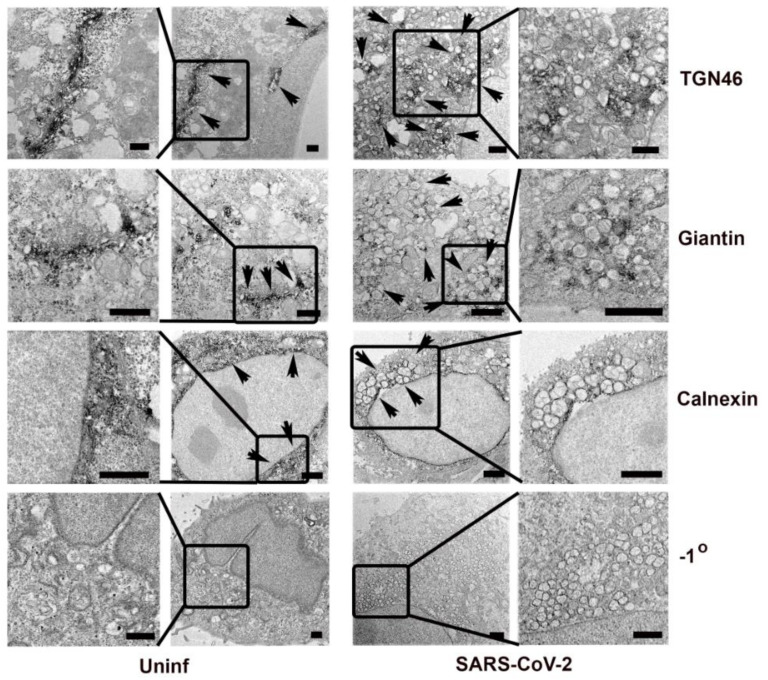
Immunoelectron microscopy localization of cellular antigens in Vero E6 cells fixed at 24 h post-infection for HRP-DAB staining. Dark reaction product indicates localization of the antigen. Shown are the Golgi specific markers TGN46 and giantin, as well as the ER marker calnexin. A panel showing negative staining in the absence of the primary antibody is shown (−1°). Arrowheads indicate regions showing HRP-DAB labeling. Delineated regions of interest in the uninfected and infected cells are enlarged in the leftmost and rightmost columns, respectively, to show detail. Bars = 1 μm.

To confirm the presence of Golgi and ER markers associated with the SARS-CoV-2 replication complex at an ultrastructural level, we used immunoelectron microscopy with HRP-DAB detection to localize TGN46, giantin, and calnexin (Figure 8). The Golgi markers were dispersed irregularly throughout the cytosol and not apparently specifically enriched on the replication organelle. The ER marker, calnexin, was observed on or around the characteristic double membrane vesicles typical of coronavirus replication organelles.

### 3.5. Viral Proteins Responsible for Golgi Disruption

**Figure 9:**
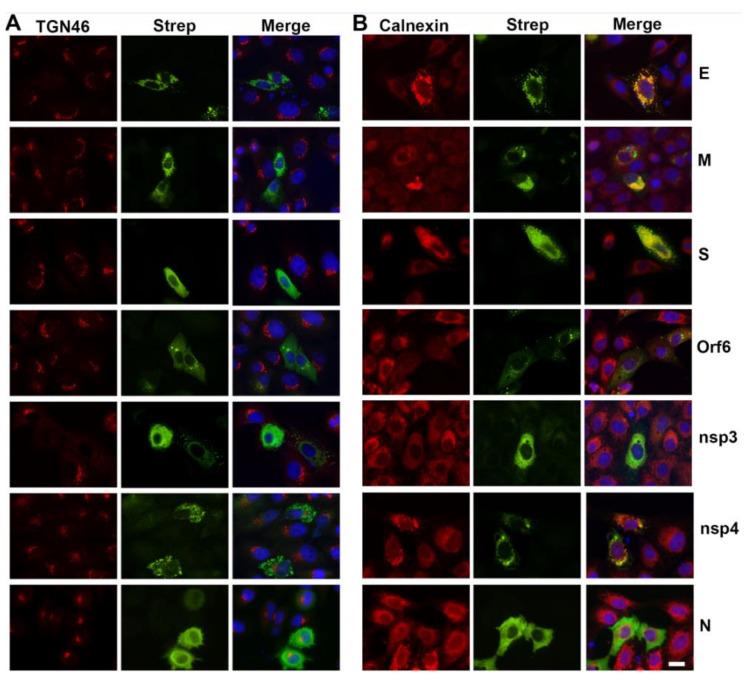
Effects of individual SARS-CoV-2 protein expression on cellular organization in transfected cells. Results are shown from Vero E6 cells transfected with epitope (Strep)-tagged E, M, S, Orf6, nsp3, nsp4, or N proteins (green). Golgi and ER architecture were monitored by labeling with anti-TGN46 (**A**) or anti-calnexin (**B**), respectively (red). Merged images are counterstained with DAPI (blue). Note the fragmentation of the Golgi apparatus by E, M, S, Orf6, or nsp3 expression. Note also changes in ER structure by all except the N protein. Bar = 10 μm.

In an effort to identify specific viral proteins that might be responsible for disruption of the Golgi apparatus, we transfected Vero cells with an expression library of SARS-CoV-2 proteins [4]. Interestingly, five of the expressed proteins, M, S, E, Orf6, and nsp3, caused dispersal of the Golgi apparatus (Figure 9). Many of these also showed association with, or reorganization of, the ER. The structural proteins, S, E, and M, as well as nsp4 were associated with large structures that generally overlapped with ER labeling. Nsp3 associated with the ER but did not display distinct condensation of the structure although it changed the organization such that it appeared more evenly distributed. Orf6 formed punctate spherical structures that were not directly associated with the ER marker calnexin. Overall ER labeling in the Orf6 transfected cells was greatly reduced.

Each of the membrane associated structural proteins, S, M, and E, formed distinct structures in transfected cells and caused dispersal of the Golgi apparatus (Figure 9). In addition, Orf6 and nsp3 also caused disruption of the Golgi, although, the N protein, nsp2, nsp4, nsp5, nsp7, nsp12, Orf7b, Orf8, Orf9b, Orf9c, and Orf10 proteins did not cause Golgi disruption (Data not shown). Transfections with the remainder of the genes expressed (Orf3b, nsp9, nsp13) were too inefficient to permit analysis.

The effects of specific SARS-CoV-2 proteins on ER structure was also examined. Each of the viral proteins (S, E, M, Orf6, and nsp3) that disrupted the Golgi apparatus also caused unique rearrangements of the ER. In addition, nsp4 associated with and caused condensation of some regions of the ER.

**Figure 10:**
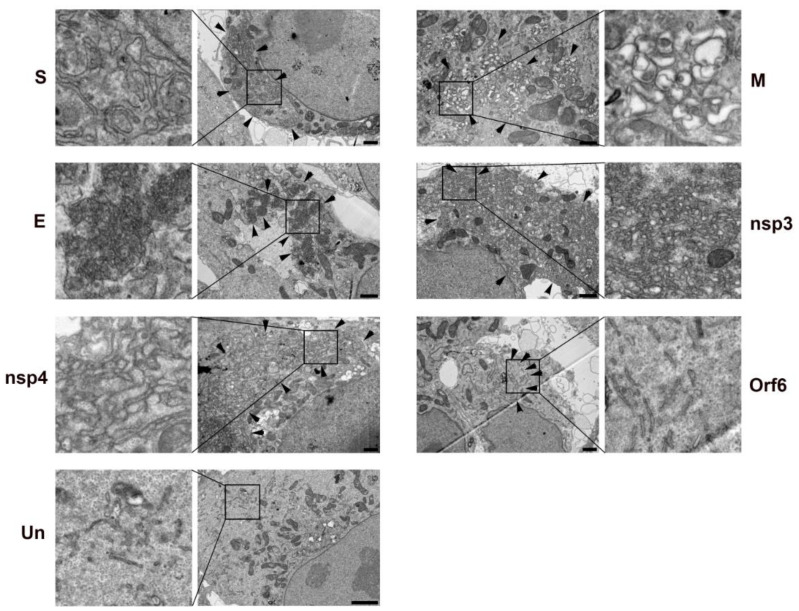
Correlative Light and Electron Microscopy (CLEM) to examine the ultrastructure of transfected cells expression Table 2. E, M, S, Orf6, nsp3, or nsp4 proteins. Transfected cells were immunolabeled with an anti-Strep tag antibody and DyLight_488_ secondary antibody. Transfected cells were identified, localized and selected for processing for TEM. Selected cells are shown in Supplementary Figure S4. Each of the six viral proteins induces a unique change in cellular membrane structure. An un-transfected cell (Un) is shown for comparison. Arrowheads indicate regions enriched in SARS-CoV-2 protein induced membrane reorganization. Delineated regions of interest in the transfected cells are enlarged in the leftmost and rightmost columns to show detail. Bars = 1 μm.

Because transfection efficiencies varied dramatically between the different constructs, we used Correlative Light and Electron Microscopy (CLEM) to identify transfected cells following immunofluorescent labeling (Figure S4). The use of CLEM provides high confidence that the observed cells are transfected with the protein of interest and has proven a powerful tool in the assessment of roles of individual viral proteins [26,27]. Individual transfected cells were processed for transmission electron microscopy (Figure 10). Each of the viral proteins with effects on Golgi or ER structure displayed unique membrane rearrangements. Expression of the Membrane (M) glycoprotein resulted in the membrane structures remarkably similar to DMVs induced by SARS-CoV-2 infection although they appeared slightly smaller than observed in the mature replication organelle. The non-structural proteins, nsp3 and nsp4, from other coronaviruses including SARS-CoV-1, MERS-CoV, and mouse hepatitis virus (MHV) had previously been described as double membrane vesicles when expressed in mammalian cells [28,29,30]. In our analysis of SARS-CoV-2 nsp3 and nsp4, we observed what appeared ultrastructurally to be tightly compacted, swollen ER forming membrane whorls. The structures induced by nsp3 appeared to be somewhat more compacted than those induced by nsp4. The spike (S) protein induced compacted whorls of ER and the envelope (E) glycoprotein produced dense, almost spherical structures. Orf6, which caused an apparent reduction in calnexin signal in transfected cells, seemed to elicit a fragmentation of the ER. How these, and potentially other, viral proteins cooperate to create the replication organelle is not fully understood.

### 3.6. Requirement for SARS-CoV-2 Dissolution of the Golgi Apparatus for Replication

**Figure 11:**
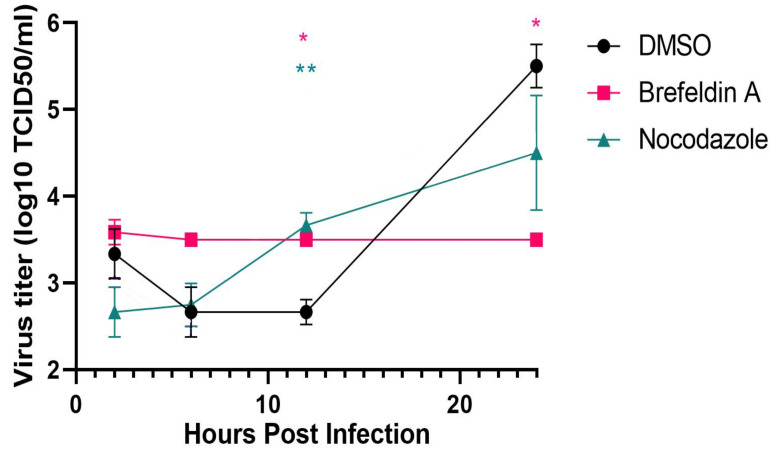
Effects of Golgi fragmentation induced by the inhibitors brefeldin A or nocodazole on SARS-CoV-2 replication. Vero E6 cells were infected with SARS-CoV-2 at 0 h and the brefeldin A (10 μM final), nocodazole (10 μg/mL), or DMSO carrier (0.4 μl/mL) added at 2 h post infection. Supernatants were collected at 2, 6, 12 and 24 h post-infection and titered by end-point dilution. Note: *p < 0.05 DMSO: Brefeldin A in pink and **p < 0.01 DMSO: Nocodazole in green.

We tested two inhibitors known to disrupt Golgi structure and function, brefeldin A, an inhibitor of the guanylate binding protein ARF1, and nocodazole, which disrupts microtubules and Golgi organization. Vero cells were infected with SARS-CoV-2 at an M.O.I. of approx. 1. At 1 h post-infection, the medium was replaced with or without brefeldin A (10 μM) or nocodazole (10 μg/mL). The carrier, DMSO, was added to a final concentration of 0.4 μl/mL in the negative controls. At 2, 6, 12, and 24 h post-infection, the supernatants were removed and viral titers determined by limiting dilution on fresh Vero cell monolayers (Figure 11). Parallel cultures were fixed at the same times for immunofluorescence analysis of effects upon Golgi structure. Treatment with brefeldin A showed no change in viral titer over the 24 h observation period resulting in almost 2 logs fewer TCID_50_/_mL_ relative to the negative control (*n* = 3; *p* < 0.05). Nocodazole treatment resulted in approx. a one log decrease in TCID_50_/_mL_ although the results were not statistically significant at 24 h post-infection.

The effects of the inhibitors were confirmed by immunofluorescence using antibodies against the Golgi marker, giantin (Figure S5). In untreated cultures, the Golgi apparatus was dispersed in infected, but not uninfected cells as described above. In brefeldin A treated cultures, the Golgi apparatus was dispersed in all cells whether infected or not, with no apparent morphological differences noted. Nocodazole treatment, however, elicited different effects in infected vs. non-infected cells. In SARS-CoV-2 infected cells, the Golgi apparatus was dispersed diffusely throughout the cytosol but in the uninfected cells, the Golgi was dispersed but displayed a discrete punctate pattern that was disseminated throughout the cytosol. Staining for β-tubulin similarly confirmed the disruption of microtubules (Figure S6).

## 4. Discussion

Multiple immunolabeling techniques incorporating both light and electron microscopy were utilized to examine the cellular origins of the SARS-CoV-2 replication organelle. Host membranes associated with the coronavirus replication complex are frequently identified as ER in origin based upon ultrastructure alone [30]. Several studies, including the present, using IFA or IEM confirm ER components in association with the coronavirus replication complex [8,11,19,20,22,31,32,33]. In addition, we confirm recent reports [18,34] showing disruption of the Golgi apparatus by SARS-CoV-2. We further provide evidence suggesting Golgi-derived lipid incorporation into the replication organelle. The means of viral dispersal of the Golgi apparatus is unknown, but the mechanism appears critical to the virus as dispersal of the Golgi with a chemical inhibitor, brefeldin A, is inhibitory to viral replication. In addition, we demonstrate that multiple SARS-CoV-2 proteins individually induce fragmentation of the Golgi apparatus. The proteins are M, S, E, Orf6, and nsp3. Each of these, as well as nsp4, also induce unique rearrangements of the ER. These observations provide a foundation for future work delineating how the various viral components might cooperate to subvert host membrane function to establish a viral replication complex. Such knowledge may help identify unique targets for chemotherapeutic intervention.

The ER has long been suspected of being a major component of the coronavirus replication organelle. Although ultrastructure alone has frequently been used to identify ER connections to the replication organelle, multiple ER specific markers have confirmed the ER origin of the replication organelle [19,20,22]. We similarly find that the ER marker, calnexin, showed the best and most specific association of the ER with the replication organelles as identified by dsRNA staining. The ER appeared contracted in infected cells and retracted from the cytosol to concentrate near the replication organelle. By immunoelectron microscopy, the outer layer of vesicles containing multiple internal vesicles displayed the ER marker but membranes of the internal vesicles did not. Immunoperoxidase staining for immunoEM necessitates the use of a mild detergent, saponin, to maintain cellular architecture. We cannot definitively comment on the composition of the internal membranes at this time since a simple failure of the mild detergent to permeabilize the outer layer to antibodies could explain the absence of internal labeling.

In addition to the compaction of the ER, one of the more notable effects on cellular structure was fragmentation of the Golgi apparatus. This dispersal of the Golgi was first detectable by 6 h post-infection and complete by 12–24 h post-infection. Ultrastructurally, the Golgi markers, TGN46 and giantin, were interspersed apparently randomly in regions of the replication organelle whereas calnexin uniformly labeled the outer membrane of the DMVs. Interestingly, NBD sphingomyelin derived from NBD-ceramide, appeared to label the replication organelle suggesting that Golgi derived lipids may distribute differently than the protein markers. The viral mechanisms responsible for Golgi fragmentation are unknown but multiple viral proteins appear to be involved.

Multiple individual coronavirus proteins have been found to associate with various cellular markers or induce changes in membrane architecture. Our primary interest was in the identification of the SARS-CoV-2 protein responsible for the dissolution of the Golgi apparatus. Surprisingly, five individual proteins, M, S, E, Orf6, and nsp3, expressed in Vero cells induced fragmentation of the Golgi. These same five proteins, as well as nsp4, also induced changes to ER organization. Each of these proteins expressed individually resulted in unique effects upon cellular membrane structure. Nsp3 and nsp4 induced formation of highly compacted membrane whorls that appeared to be of ER origin. These structures may be equivalent to the disordered membrane bodies or maze-like bodies induced by the individual expression of SARS-CoV-1 nsp3 or 4, respectively [30].

Co-expression of nsp3, 4, and 6 of SAR-CoV-1 [30] or nsp3 and 4 of MERS-CoV [29] or MHV [28] had previously been shown to be sufficient to induce formation of double membrane vesicles reminiscent of the replication organelle. We observed distinct changes in ER morphology after expression individually of both nsp 3 and nsp 4 although we did not attempt dual expression. The most striking resemblance to the coronavirus replication organelle was produced by expression of the SARS-CoV-2 M glycoprotein. These DMVs were somewhat smaller than those induced by the native virus. The M protein was of initial interest since it was observed in association with the Golgi apparatus as soon as expression was detected although the association seemed to dissipate as the Golgi dispersed. Previous studies had similarly found association of the SARS-CoV-1 and MERS-CoV M protein with the Golgi apparatus [22,35,36]. Clearly, multiple coronavirus proteins contribute to the dramatic membrane rearrangements that result in replication organelle development. Defining how these and other coronavirus proteins function cooperatively to produce the replication organelle remains a challenge.

An interaction map of 26 of the 29 SARS-CoV-2 proteins with eukaryotic interacting protein partners identified 332 high-confidence interactions between SARS-CoV-2 and human proteins [4]. Several of these interactions involve host proteins potentially involved in Golgi structure or membrane trafficking, thus there may be additional coronavirus proteins participating in creation of the replication organelle.

We observed that brefeldin A strongly inhibited SARS-CoV-2 progeny production. Similarly, brefeldin A has been shown to inhibit mouse hepatitis coronavirus (MHV) replication [19,21,37]. In addition, siRNA-mediated knockdown of cellular target of brefeldin A, the guanine nucleotide exchange factor GBF1 or its effector, the small GTPase ARF1, also were inhibitory to MHV replication [37]. Brefeldin A treatment causes fragmentation of the Golgi apparatus and retrograde trafficking of resident Golgi proteins back into the ER. The effect of this is an inhibition of anterograde vesicular traffic from the Golgi apparatus. Disruption of microtubules by nocodazole also causes fragmentation of the Golgi apparatus [38]. Nocodazole treatment causes a modest but not statistically significant decrease in SARS-CoV-2 progeny production. The mechanism of viral disruption of the Golgi apparatus is unclear although several viral proteins can produce this effect. Inhibition of Golgi function by brefeldin A is inhibitory to SARS-CoV-2 replication. Whether the fragmentation of the Golgi by brefeldin A directly causes the reduction in viral replication is possible, perhaps by inhibition of some early viral function. However, a more likely possibility is that the GBF1 and/or ARF1 may be critical to some aspect of viral replication.

With large numbers of coronaviruses in nature and the potential for further cross-species transmission, it is likely that we will see future outbreaks of human coronavirus disease. An improved understanding of viral mechanisms used to create favorable conditions for replication may suggest novel targets for chemotherapeutic intervention.

## Data Availability

Data is contained within the article or supplementary material.

## Supplementary Materials

The following are available online at www.mdpi.com/xxx/s1,Figure S1: Temporal expression of SARS-CoV-2 structural proteins. Figure S2: Time course of the fragmentation of the Golgi apparatus observed during SARS-CoV-2 infection of Vero E6 cells. Figure S3: Fragmentation of the Golgi apparatus observed during SARS-CoV-2 infection of ACE2-expressing A549 human lung epithelial cells at 24 h post-infection. Figure S4: Transfected Vero cells on gridded coverslips expressing the SARS-CoV-2 S, M, E, nsp3, nsp4, or Orf6 proteins for correlative light and electron microscopy (CLEM). Figure S5: Effects of brefeldin A and nocodazole on Golgi structure. Figure S6: Effects of brefeldin A and nocodazole on microtubule organization. Video S1: Reconstruction of SARS-COV-2 infected Vero cell from FIB-SEM data.
