# Supplementary material for: Disruption of the Golgi Apparatus and Contribution of the Endoplasmic Reticulum to the SARS-CoV-2 Replication Complex
Source: Viruses. 2021 Sep 9;13(9):1798. doi: 10.3390/v13091798 (PMC8473243; doi:10.3390/v13091798)
Supplement: Supplementary file 1 [file viruses-13-01798-s001.zip › viruses-1340797-suppl-conv/viruses-1340797-suppl-conversion.pdf]

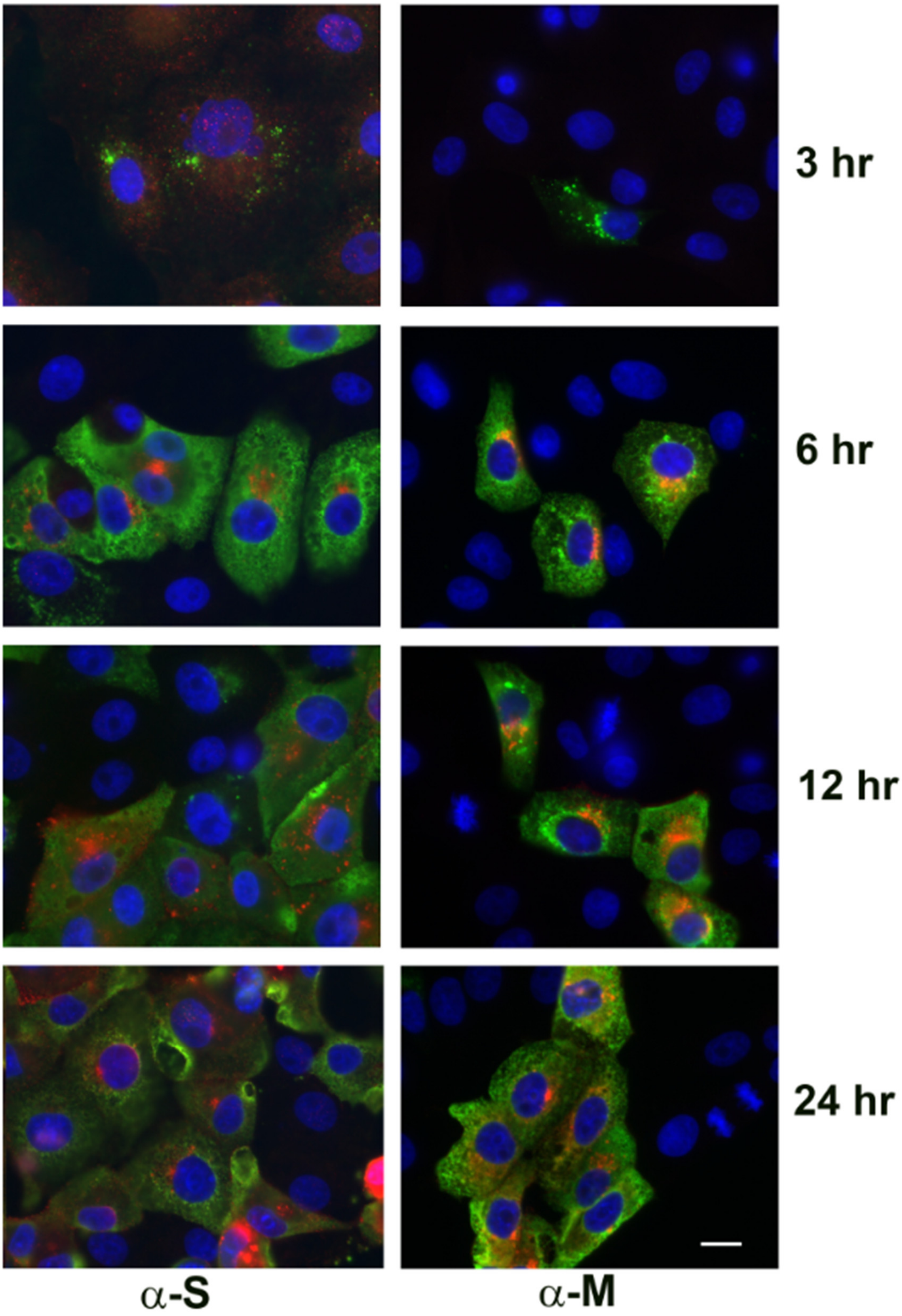

Figure S1. Temporal expression of SARS-CoV-2 structural proteins. The spike protein (S) and the membrane glycoprotein (M) are shown in red. The nucleocapsid protein (N) is the first detected and is shown in green. Nuclei are counterstained with DAPI (blue). Bar = 10 µm.

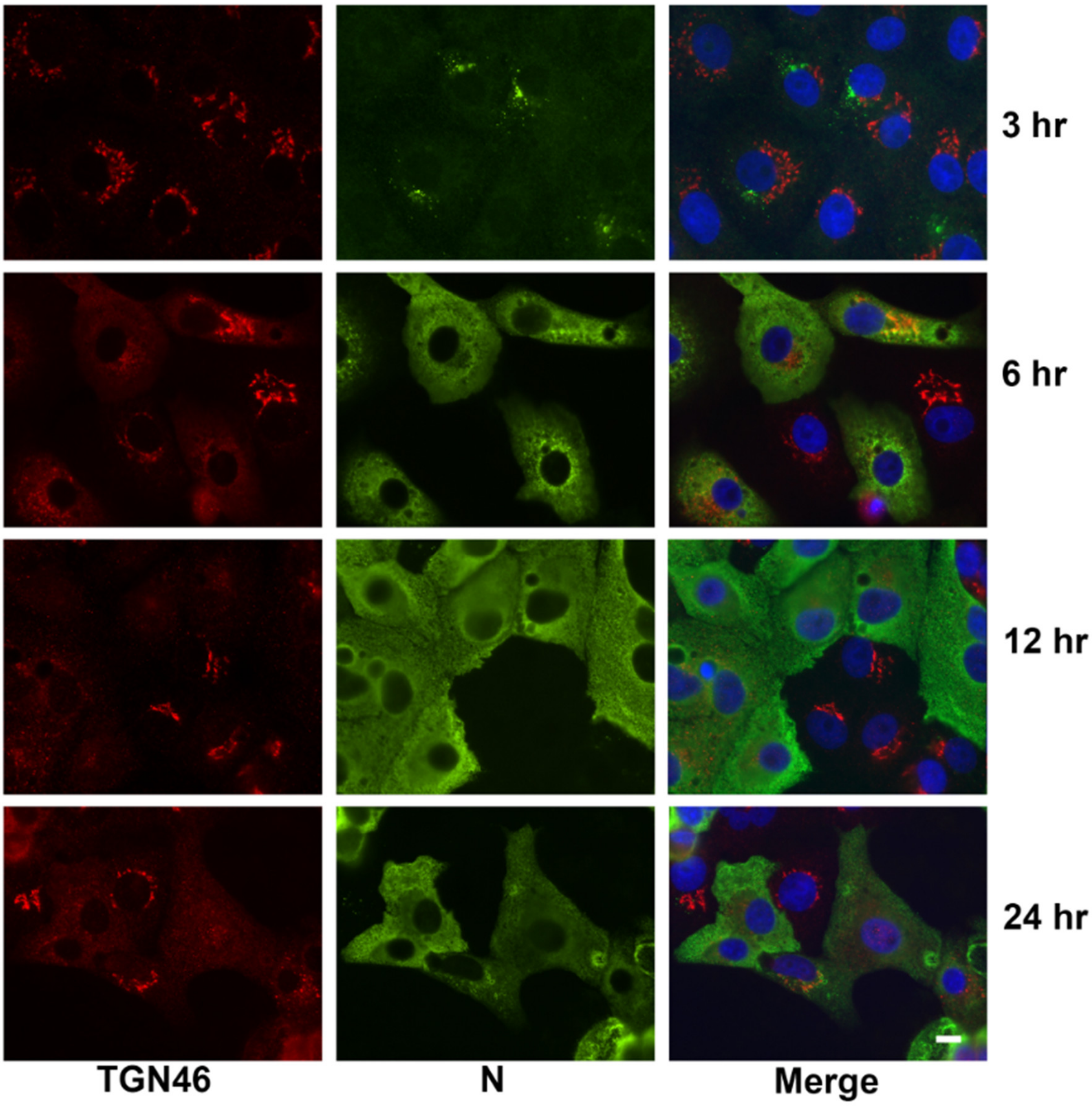

Figure S2. Time course of the fragmentation of the Golgi apparatus observed during SARS-CoV-2 infection of Vero E6 cells. Infected cells are identified by labeling against the nucleocapsid protein (green). The Golgi apparatus is disrupted by 6 h post-infection and completely dispersed by 12 h. Nuclei are counterstained with DAPI (blue) in the merged images. Bar = 10 μm.

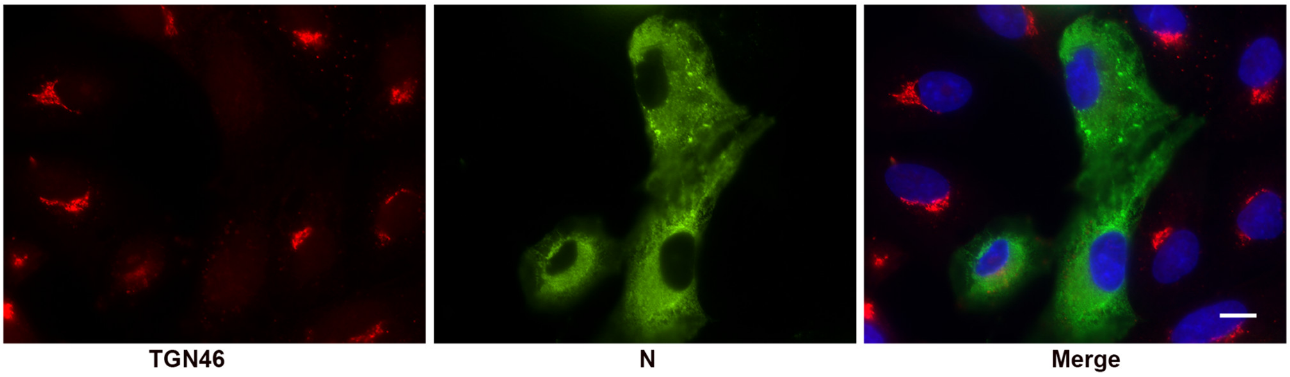

Figure S3. Fragmentation of the Golgi apparatus observed during SARS-CoV-2 infection of ACE2-expressing A549 human lung epithelial cells at 24 h post-infection. Infected cells are identified by labeling against the nucleoprotein (N) (green). Nuclei are counterstained with DAPI (blue) in the merged images. Bar = 10 μm.

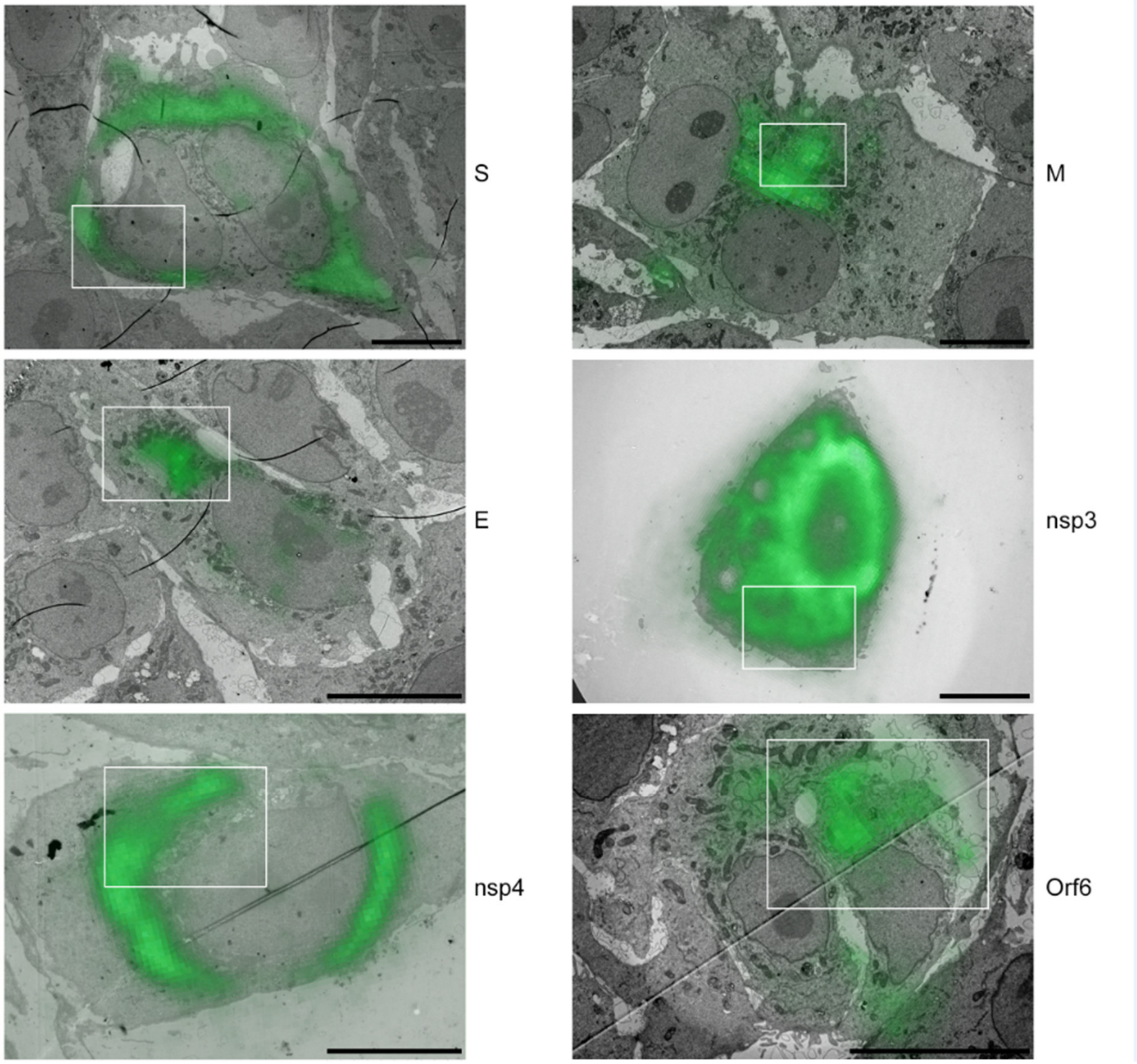

Figure S4. Transfected Vero cells on gridded coverslips expressing the SARS-CoV-2 S, M, E, nsp3, nsp4, or Orf6 proteins for correlative light and electron microscopy (CLEM). Transfected cells were identified by indirect immunofluorescence using an anti-strep tag antibody. Overlays of the fluorescent images over the micrographs are shown. Selected cells were trimmed and sectioned for TEM.

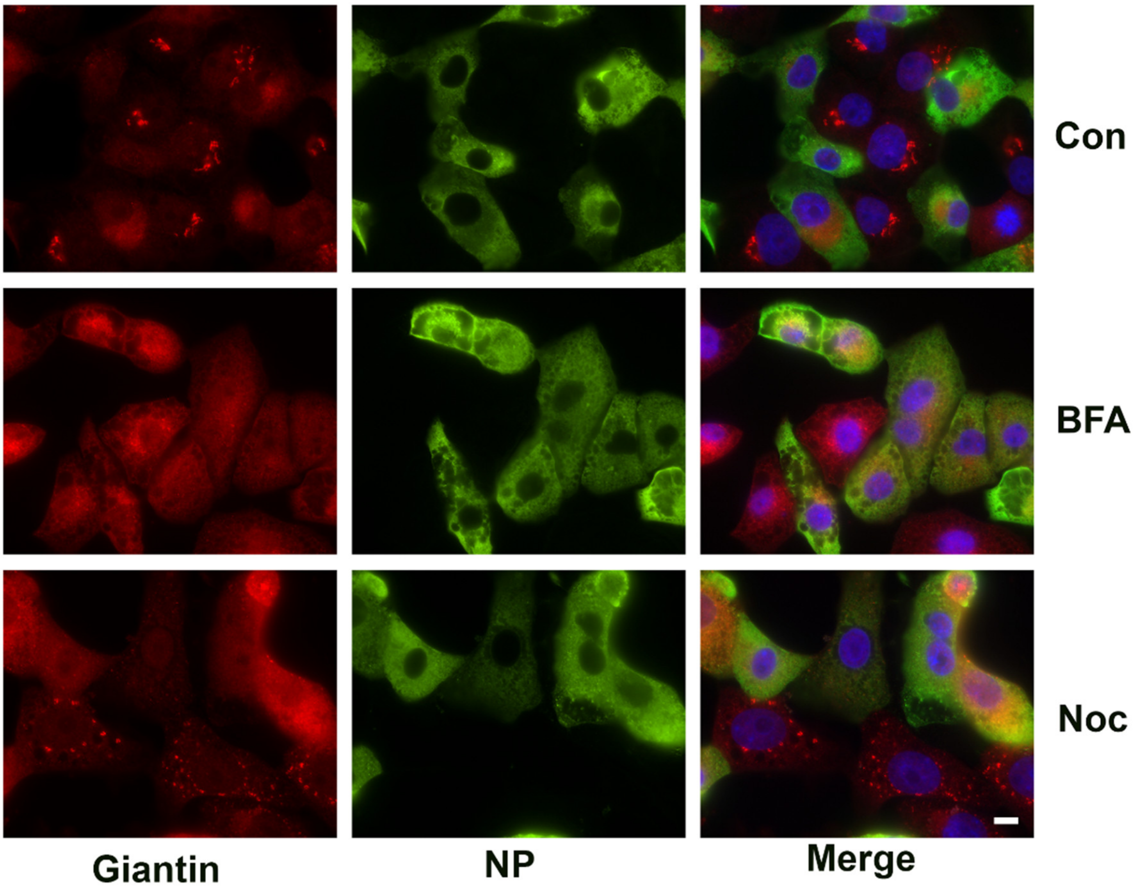

Figure S5. Effects of brefeldin A and nocodazole on Golgi structure. SARS-CoV-2 infected cells were treated with DMSO carrier alone as a negative control (Con) or with brefeldin A (BFA) or nocodazole (Noc). Infected cells were identified by labeling with anti-nucleoprotein (NP) (green) and the Golgi apparatus with anti-Giantin (red). Note that in the controls, the Golgi apparatus is dispersed only in the infected cells. In the brefeldin treated cells, the Golgi is dispersed in both infected and uninfected cells. In the nocodazole treated cultures, the Gogli apparatus is dispersed into a discrete punctate, vesicular pattern in the unfiected cells but completely dispersed in the infected cells. Nuclei are counterstained with DAPI (blue) in the merged images. Bar = 10 μm.

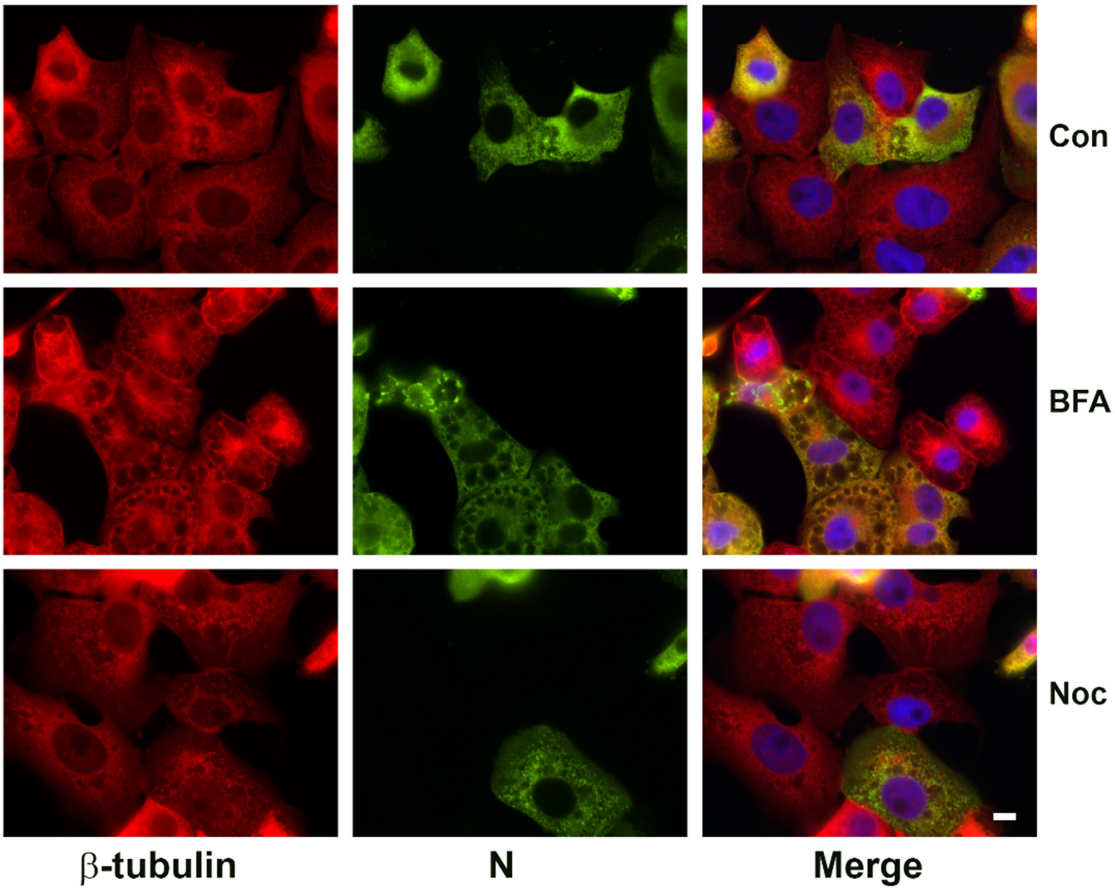

Figure S6. Effects of brefeldin A and nocodazole on microtubule organization. SARS-CoV-2 infected cells were treated with DMSO carrier alone as a negative control (Con) or with brefeldin A (BFA) or nocodazole (Noc). Infected cells were identified by labeling with anti-nucleocapsid protein (N) (green) and microtubules with anti-tubulin (red). Nuclei are counterstained with DAPI (blue) in the merged images. Bar = 10 μm.
